# Digital versus analogue record systems for mass casualty incidents at sea—Results from an exploratory study

**DOI:** 10.1371/journal.pone.0234156

**Published:** 2020-06-05

**Authors:** Esther Henning, Mustafa Sinan Bakir, Lyubomir Haralambiev, Simon Kim, Stefan Schulz-Drost, Peter Hinz, Thomas Kohlmann, Axel Ekkernkamp, Denis Gümbel

**Affiliations:** 1 Department of Trauma, Reconstructive Surgery and Rehabilitation Medicine, University Medicine Greifswald, Greifswald, Germany; 2 Department of Trauma and Orthopaedic Surgery, BG Klinikum Unfallkrankenhaus Berlin gGmbH, Berlin, Germany; 3 Department of Trauma Surgery, Helios Kliniken Schwerin, Schwerin, Germany; 4 Institute for Community Medicine, Section Methods in Community Medicine, University Medicine Greifswald, Germany; University Hospital Zurich, SWITZERLAND

## Abstract

**Objective:**

Mis-triage may have serious consequences for patients in mass casualty incidents (MCI) at sea. The purpose of this study was to assess outcome, reliability and validity of an analogue and a digital recording system for triage of a MCI at sea.

**Methods:**

The study based on a triage exercise conducted with a cross-over-design. Forty-eight volunteers were presented a fictional MCI with 50 cases. The volunteers were randomly assigned to start with the analogue (Group A, starting with the analogue followed by the digital system) or digital system (Group B, starting with the digital followed by the analogue system). Triage score distribution and agreement between the triage methods and a predefined standard were reported. Reliability was analysed using Cronbach’s Alpha and Cohen’s Kappa. Validity was measured through sensitivity, specificity and predictive value. Treatment, period and carry-over-effects were analysed using a linear mixed-effects model.

**Results:**

The number of patients triaged (total: n = 3545) with the analogue system (n = 1914; 79.75%) was significantly higher (p = 0.001) than with the digital system (n = 1631; 67.96%). A trend towards a higher percentage of correct triages with the digital system was observed (p = 0.282). Ratio of under-triage was significantly smaller with the digital system (p = 0.001). Validity measured with Cronbach’s Alpha and Cohen’s Kappa was higher with the digital system. So was sensitivity (category; green: 80.67%, yellow: 73.24%, red: 83.54%; analogue: green: 93.28%, yellow: 82.36%, red: 94.04%) and specificity of the digital system (green: 78.07%, yellow: 63.75%, red: 66.25%; analogue: green: 85.50%, yellow: 79.88%, red: 91.50%). Comparing the predictive values and accuracy, the digital system showed higher scores than the analogue system. No significant patterns of carry-over-effects were observed.

**Conclusions:**

Significant differences were found for the number of triages comparing the analogue and digital recording system. The digital system has a slightly higher reliability and validity than the analogue triage system.

## Introduction

A Mass Casualty Incident (MCI) is an overwhelming event with more patients at the same time than locally available resources can manage using routine procedures. It requires exceptional emergency response and additional, extraordinary support [1]. MCI pose a major challenge for medical personnel [2,3]. A mismatch between the number of patients and the available medical, technical and administrative resources are a characteristic of a MCI [1–4].

Ashore, established algorithms are initiated to enable the best possible care for all patients involved in a MCI [4]. The primary objective is to use the available resources efficiently to ensure the survival of as many victims as possible [5,6]. A central component for coping with a MCI is the triage [5–7]. The purpose of triage is to prioritize treatment to use the existing medical, material and human resources efficiently following the principle of "giving best possible care for as many patients as possible". The use of triage algorithms allows the identification of critically injured patients and the determination of the need for immediate treatment [7]. There are many triage algorithms available. One of the most commonly used preclinical triage algorithms is STaRT (simple triage and rapid treatment). With the STaRT- algorithm, the victims can be divided into four categories: red (life-threatening injuries, immediate treatment required), yellow (severe injuries, treatment can be delayed) or green (minor injuries) and black (deceased/expectant) [8]. Due to insufficient training, injury severity is often misjudged [5].

To optimize care during a MCI ashore, electronic support systems with portable computers and tablets have been tested for routine triage and transportation organization [9]. In addition to the support function through an algorithm-based triage and transport prioritization for the rescue workers involved, digital systems can support the planning and decision-making process and facilitate evaluation [10].

A further central component for coping with this situation is the triage exercise. Such exercises have been established in different ways to train physicians, paramedics, nurses and further involved persons [11–13].

MCIs at sea occur rarely, but with severe consequences, due to the location and the remoteness from professional emergency staff in most cases [4]. Further aspects, e.g., time and weather, may influence these conditions [14]. Resulting in a delayed rescue in medically and operationally extremely precarious situations [4]. For this reason, the ability of the ship’s crew to assess the number and severity of the injured passengers plays a central role [14].

To support the ship crew during a MCI at sea, the analogue Lübecker Recording System for Major Accidents (LüDoG) was developed for such situations [15]. It defines the documentation of triage results, medical care given and an overview regarding patient prioritization. The LüDoG consists of a documentation bag with coloured patient attachments of the triage categories green, yellow, red and grey (aligned to the STaRT-algorithm) and an overview documentation system. The overview documentation comprises the triage, treatment place, delivery room and hospital place. The triage documentation includes the time, diagnosis and result of the triage for each patient. At the same time, a possible transport priority and the preferred mode of transport can be documented. The document "treatment place" lists the individual injuries, triage times, diagnoses, transport priorities, transport vehicle, and destinations of the transports as well as the arrival and departure times [15]. Therefore, the LüDoG system corresponding to many other triage documentation systems, integrating additional information about transport vehicles which also includes different types of ships and a German and English translation. For medical documentation, more specified information can easily be filled out using predefined medical conditions and predefined treatment possibilities. Currently, LüDoG is the only analogue recording system for triage at sea in the German-speaking area.

For digital triage recording systems at sea have previously not been available, the “Transport organization for patients at sea” (TOPaS) Client, a digital tablet-based triage system, has recently been developed, especially for the use onboard. The system guides the users through the documentation process. An additional support function can be used to suggest the triage category, transport priority and necessary transport vehicle. Comparable to the LüDoG system, the TOPaS-Client correspond to many other triage documentation systems, which includes similar additional information as the LüDoG system. Additional to the analogue system the TOPaS-Client includes an intuitive instructions for laypeople, a multifocal screening for several teams with electronically-coupled bundling of the results as well as real-time connection to a coordination point (at sea or on land). The usability for nautical officers has been evaluated through a feedback mechanism of nautical officers. The difference between the analogue and digital system are compared in Table 1.

**Table 1:** Differences between TOPaS and LüDoG.

	TOPaS	LüDoG
**Kind of System**	Digital	analogue
**Algorithm**	STaRT (possible to choose between SALT, STaRT, mSTaRT, mSTaRT+, TRIAGE SIEVE, CARE FLIGHT)	STaRT
**Documentation**		
Personal information	yes	yes
Triage	yes	yes
Specified condition	yes	yes
Diagnosis	yes	yes
Treatment	yes	yes
Transport priority	yes	yes
Transport destination	yes	yes
**Support function**		
Suggestion triage category	yes	no
Suggestion transport priority	yes	no
**Electricity**	necessary (battery life dependent on the tablet)	not necessary
**Waterproof**	limited	limited
**Language**	English, German	English, German

Furthermore, the degree of first aid knowledge is an important factor. Regarding the International Convention on Standards of Training, Certification and Watch keeping for Seafarers (STCW), every person working on a ship should have a specified degree of first aid knowledge [16]. Depending on the type of ship, number of crew members and number of passengers as well as the duration of the sea journey, different staff with medical education are required onboard. Physicians are only required for a journey of more than three days and with 100 or more people onboard [16]. If there is no physician onboard, the captain is responsible for medical care. The captain can delegate this task to a nautical officer [17]. The medical training as nautical officers consists of 80 hours of basics in shipping medicine and extended first aid. In addition, a two-week internship in a hospital is required for German nautical staff [18].

Even though there is no mandatory triage training, first training institutions in Germany are already discussing the implementation of triage exercises in the education of navigation officers.

The present study aimed to evaluate the outcome and compare the reliability and validity of analogue versus digital triage for the practical use and training setting.

## Materials and methods

The quantitative analysis is based on a triage exercise conducted with a cross-over-design. The Ethics Committee Greifswald (registry number BB 009/19) approved the study. Informed consent was obtained from all participants prior to triage scenario introduction. The Ethics Committee Greifswald agreed with verbal consent.

For the triage exercise, a scenario was created involving a fire on a Roll-On-Roll-Off-Passenger-ferry. These ferries are designed to carry wheeled cargo and passengers. Fifty fictional patients (‘vignettes’) were created for the triage including basic parameters, such as biometric data, age, gender, ABC (airway, breathing, and circulation) information and injury of the patients. The fictional patients were developed for a fire scenario onboard. Diagnostic parameters such as blood pressure, oxygen saturation and respiratory rate were used which are content of the medical education of nautical officers and therefore possible to be generated and evaluated by them. The vignettes were printed on standard paper. The simulated patients were classified according to the following distribution key: 20% red, 30% yellow and 50% green. To determine the correct triage category, the patients were triaged by three independent emergency physicians, defining a gold standard. Each patient was given a code, including number and triage category. Fifty volunteers aged 18 to 70 years participated in the triage exercise, recruited via written call. A detailed description of the participants is shown in Table 2.

**Table 2:** Study sample and sociodemographic characteristics of the study group in total, Group A and B.

	Total (n = 48)	Group A (n = 24)	Group B (n = 24)
**Age**			
Mean (SD)	25.8 (3.5)	26.0 (2.7)	25.7 (4.2)
Range	20–33	20–30	20–33
**Sex, n (%)**			
Female	31 (64.6)	15 (62.5)	14 (58.4)
**Current status, n (%)**			
Students	45 (93.7)	22 (91.7)	23 (95.9)
Employed	3 (6.3)	2 (8.3)	1 (4.1)
**Work experience (in years)**			
Mean (SD)	3.8 (4.0)	2.4 (3.4)	6.1 (4.0)
Range	0–11	0–11	0–10
**Nautical experience, n (%)**	2 (4.2)	1 (2.1)	1 (2.1)

About 27.1% of the participants had extended experience in the medical field through internships in the area of nursing, paramedics, representing the minority of the ship crew with extended medical knowledge and experience at merchant ships. To simplify the analysis, each vignette contained a triage code. Due to the decoding of the triage codes, the data sets of two participants couldn’t be used for the further analysis.

After a short verbal briefing on the exercise, triage systems, sample patients and scenario, the participants were randomly assigned (simple and unrestricted randomization) to two equal groups. One group started with the analogue system followed by the digital system (Group A—25 participants), the other group started with the digital followed by the analogue system (Group B—25 participants). The main task consisted in the triage of the sample-patients, documenting the results with the respective recording system and marking the sample-patients in accordance with the triage-category within 45 minutes for each triage system. For the analogue triage the participants received the sample patients and the LüDoG system. For digital documentation, the participants received the sample patients, a tablet with the TOPaS client and coloured paper in the three colours grey, red, yellow and green (analogous to the LüDoG).

Between the two triage exercises there was a time frame of 30 minutes. After the exercise, the participants completed a questionnaire with personal information including age, sex, employment status, working experience as well as medical and nautical background.

The primary outcome indicator was the number of completed triages in the predetermined time and their correctness compared to the gold standard. Over-triage and under-triage were calculated based on the defined triage categories. Critical under-triage was defined as two triage categories lower than the gold standard. Descriptive analysis was used to show the triage results (Mean, Standard Deviation—SD, Ratio). Because normal distribution of data could be assumed after inspection of histograms and boxplots, the t-test for paired samples was used comparing the analogue and digital results as well as analogue 1 versus digital 2 and digital 1 versus analogue 2. For the comparison of Group A versus Group B, the analogue 1 and analogue 2 results as well as digital 1 and digital 2 results the t-test for unpaired samples was used. The significance level was defined as p<0.05.

To investigate the reliability and consistency of the key indicators, Cronbach’s Alpha was calculated for the measurement of the correct triage of the analogue and digital triage results [19,20]. Additionally, the unweighted (used for measuring the exact agreement in nominal and ordinal scales) and weighted kappa (allows weighting of different disagreements) for triage evaluation were used [21,2]. Unweighted and weighted Cohen’s Kappa, including confidence interval, were used as the statistical measure of interrater reliability of assessments [22,23]. Sensitivity and specificity were chosen as outcome measures in this study, as the most important diagnostic parameter in theory and teaching [24]. Furthermore, the predictive value was used to assess validity [24]. To analyse treatment, period and carryover effects, a linear regression model [25], the mixed-effects model was used.

The data were statistically evaluated using the Statistical Package for the Social Sciences (SPSS) for Windows, Version SPSS 24.0 (IBM, Armonk, New York, USA).

## Results

During the exercise, each of the 48 participants performed both triages with each of the 50 simulated patients, so that a total of 4800 triages could be conducted. In total, 73.85% (n = 3545) of the possible triages were carried out in the given time. 67.96% of the triages were performed with the digital system and 79.75% with the analogue system. Each participant triaged an average of 73.9 (SD 18.1) patients. Of these, an average of 55.2 (SD 16.7) patients were correctly triaged, 11.5 (SD 7.9) were over- and 7.0 (SD 3.4) under-triaged.

**Fig 1:**
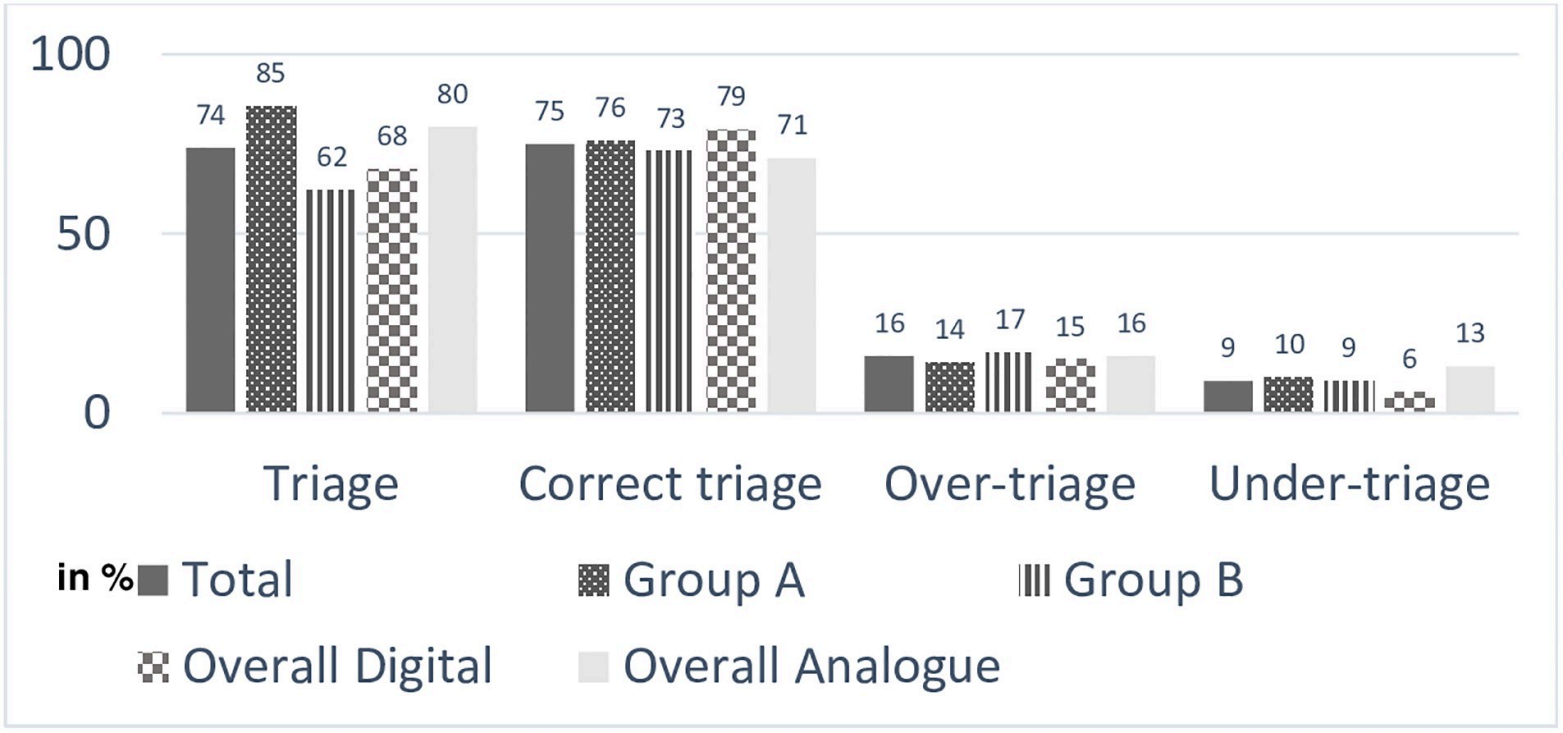
Number of triage, correct, over- and under-triage in % for all triages, Group A, B, digital and analogue. The figure display the number of triage, correct triage as well as over and under-triage in percentage conducted during the triage exercise with an analogue and a digital triage system for a mci at sea. The difference of the results are presented between the different triage Groups, the digital, the analogue, the Group A (starting with the analogue followed by the digital system) and the Group B (starting with the digital followed by the analogue system).

Fig 1 gives an overview about the descriptive results of all triages conducted in Group A and B as well as the digital and analogue results.

Using the digital system, significantly fewer triages were performed than with the analogue system (p = 0.001). The participants achieved a higher proportion of correct triages with the digital system than with the analogue system and fewer correct triages in total. The difference was not significant (p = 0.282). The over-triage rate of participants using the digital system was lower than with the analogue system. The over-triage rate did not differ significantly (p = 0.164). Under-triage was significantly less frequent with the digital system (p = 0.001). A detailed overview of the number of triages and correct triages is shown in Table 3.

**Table 3:** Number of triage and correct triage in total and divided into analogue and digital system as well as Group A and B.

**Total (Digital versus Analogue)**										
	**Total**			**Digital**			**Analogue**			**Difference Digital—Analogue**
	N	Mean triage per participant	Ratio of accomplished triage in %	N	Mean triage per participant	Ratio of accomplished triage in %	N	Mean triage per participant	Ratio of accomplished triage in %	P-value
**Triage**	3545	73.9 (SD 18.1)	73.8	1631	34.0 (SD 11.0)	68.0	1914	39.9 (SD 10.5)	79.7	<0.001*
**Correct triage**	2652	55.2 (SD 16.7)	74.8	1288	26.8 (SD 10.1)	79.0	1364	28.4 (SD 9.4)	71.3	0.282
**Over-triage**	554	11.5 (SD 7.9)	15.6	251	5.2 (SD 5.0)	15.4	303	6.3 (SD 4.5)	15.8	0.164
**Under-triage**	339	7 (SD 3.4)	9.6	92	1.9 (SD 1.8)	5.6	247	5.1 (SD 2.9)	12.9	<0.001*
**Total (Group A versus Group B)**										
	**Total**			**Group A (Analogue—Digital)**			**Group B (Digital—Analogue)**			**Difference Group A—Group B**
	N	Mean triage per participant	Ratio of accomplished triage in %	N	Mean Triage per participant	Ratio of accomplished triage in %	N	Mean Triage per participant	Ratio of accomplished triage in %	P-value
**Triage**	3545	73.9 (SD 18.1)	73.8	2049	42.7 (SD 8.9)	85.4	1496	31.2 (SD 10.2)	62.3	<0.001*
**Correct triage**	2652	55.2 (SD 16.7)	74.8	1556	32.4 (SD 9.2)	76.0	1096	22.8 (SD 7.7)	73.3	<0.001*
**Over-triage**	554	11.5 (SD 7.9)	15.6	295	6.1 (SD 5.7)	14.4	259	5.4 (SD 3.7)	17.3	0.516
**Under-triage**	339	7 (SD 3.4)	9.6	198	4.1 (SD 2.8)	9.7	141	2.9 (SD 2.9)	9.4	0.013*
**Group A (Analogue 1 versus Digital 2)**										
	**Group A**			**Analogue 1**			**Digital 2**			**Difference Analogue 1—Digital 2**
	N	Mean triage per participant	Ratio of accomplished triage in %	N	Mean triage per participant	Ratio of accomplished triage in %	N	Mean triage per participant	Ratio of accomplished triage in %	P-value
**Triage**	2049	42.7 (SD 10.9)	85.4	1077	44.9 (SD 9.0)	89.7	972	40.5 (SD 8.4)	81.0	0.034*
**Correct triage**	1556	32.4 (SD 9.7)	76.0	775	32.3 (SD 9.5)	72.0	781	32.5 (SD 9.1)	80.3	0.898
**Over-triage**	295	6.1 (SD 5.8)	14.4	162	6.7 (SD 5.4)	15.0	133	5.5 (SD 6.0)	13.7	0.274
**Under-triage**	198	4.1 (SD 2.8)	9.7	140	5.8 (SD 2.5)	13.0	58	2.4 (SD 1.9)	6.0	<0.001*
**Group B (Digital 1 versus Analogue 2)**										
	**Group B**			**Digital 1**			**Analogue 2**			**Difference Digital 1—Analogue 2**
	N	Mean triage per participant	Ratio of accomplished triage in %	N	Mean triage per participant	Ratio of accomplished triage in %	N	Mean triage per participant	Ratio of accomplished triage in %	P-value
**Triage**	1496	31.2 (SD 11.1)	62.3	659	27.5 (SD 9.5)	54.9	837	34.9 (SD 9.7)	69.7	0.015*
**Correct triage**	1096	22.8 (SD 8.4)	73.3	507	21.1 (SD 7.7)	76.9	589	24.5 (SD 7.5)	70.4	0.128
**Over-triage**	259	5.4 (SD 4.6)	17.3	118	4.9 (SD 3.8)	17.9	141	5.9 (SD 3.5)	16.8	0.398
**Under-triage**	141	2.9 (SD 2.8)	9.4	34	1.4 (SD 1.6)	5.2	107	4.5 (SD 3.1)	12.8	<0.001*
**Digital (Digital 1 versus Digital 2)**										
	**Digital**			**Digital 1**			**Digital 2**			**Difference Digital 1—Digital 2**
	N	Mean triage per participant	Ratio of accomplished triage in %	N	Mean triage per participant	Ratio of accomplished triage in %	N	Mean triage per participant	Ratio of accomplished triage in %	P-value
**Triage**	1631	34.0 (SD 11.0)	68.0	659	27.5 (SD 9.5)	54.9	972	40.5 (SD 8.4)	81.0	<0.001*
**Correct triage**	1288	26.8 (SD 10.1)	79.0	507	21.1 (SD 7.7)	76.9	781	32.5 (SD 9.1)	80.3	0.001*
**Over-triage**	251	5.2 (SD 5.0)	15.4	118	4.9 (SD 3.8)	17.9	133	5.5 (SD 6.0)	13.7	0.668*
**Under-triage**	92	1.9 (SD 1.8)	5.6	34	1.4 (SD 1.6)	5.2	58	2.4 (SD 1.9)	6.0	0.056
**Analogue (Analogue 1 versus Analogue 2)**										
	**Analogue**			**Analogue 1**			**Analogue 2**			**Difference Analogue 1—Analogue 2**
	N	Mean triage per participant	Ratio of accomplished triage in %	N	Mean triage per participant	Ratio of accomplished triage in %	N	Mean triage per participant	Ratio of accomplished triage in %	P-value
**Triage**	1914	39.9 (SD 10.5)	79.7	1077	44.9 (SD 9.0)	89.7	837	34.9 (SD 9.7)	69.7	<0.001*
**Correct triage**	1364	28.4 (SD 9.4)	71.3	775	32.3 (SD 9.5)	72.0	589	24.5 (SD 7.5)	70.4	0.003*
**Over-triage**	303	6.3 (SD 4.5)	15.8	162	6.7 (SD 5.4)	15.0	141	5.9 (SD 3.5)	16.8	0.254
**Under-triage**	247	5.1 (SD 2.9)	12.9	140	5.8 (SD 2.5)	13.0	107	4.5 (SD 3.1)	12.8	0.096

* = p-value <0.05

### Reliability: Cronbach’s Alpha and Cohen’s Kappa

The digital triage results (0.931) reached a higher Cronbach’s Alpha than the analogue system (0.924). For both types of kappa (unweighted, linear weighted), the digital system results obtained a higher kappa value than the results of the analogue system (Table 4).

**Table 4:** Reliability results using unweighted and linear weighted Kappa comparing the analogue and digital system as well as Group A and B.

	Unweighted Kappa (95% Confidence interval)	Linear weighted Kappa (95% Confidence interval)
**Analogue**	0.55 (0.52–0.58)	0.64 (0.62–0.67)
**Digital**	0.67 (0.64–0.71)	0.75 (0.73–0.78)
**Group A**	0.63 (0.60–0.66)	0.71 (0.69–0.74)
**Group B**	0.58 (0.54–0.62)	0.67 (0.64–0.70)

### Validity: Sensitivity, specificity, and predictive value

Compared to the analogue system, triage with the digital system achieved a higher validity in all categories (Tables 5 and 6).

**Table 5:** Sensitivity and specificity results of the digital and analogue system as well as Group A and B.

	Sensitivity (95% Confidence interval)				Specificity (95% Confidence interval)			
	Digital	Analogue	Group A	Group B	Digital	Analogue	Group A	Group B
**Red**	83.54† (79.03–87.42)	66.25 (61.36–70.89)	68.59 (63.89–73.01)	80.39† (75.49–84.69)	94.04† (92.62–95.26)	91.50 (89.98–92.85)	93.38 (92.06–94.55)	91.70 (90.00–93.20)
**Yellow**	73.24† (69.12–77.08)	63.75 (59.69–67.66)	70.94† (67.18–74.50)	64.78 (60.22–69.15)	82.36† (80.02–84.54)	79.88 (77.62–82.00)	82.44 (80.36–84.39)	79.16 (76.58–81.58)
**Green**	80.67† (77.78–83.33)	78.07 (75.28–80.69)	83.82† (81.39–86.05)	74.09 (70.79–77.21)	93.28† (91.35–94.90)	85.50 (83.13–7.64)	92.73† (91.01–94.21)	88.12 (85.62–90.33)

^†^ higher value comparing analogue versus digital as well as Group A versus B

**Table 6:** Predictive value and accuracy results of the digital and analogue system as well as Group A and B.

Positive predictive value (95% Confidence interval)				Negative predictive value (95% Confidence interval)				Accuracy (95% Confidence interval)			
Digital	Analogue	Group A	Group B	Digital	Analogue	Group A	Group B	Digital	Analogue	Group A	Group B
77.52† (73.45–81.13)	67.09 (63.02–70.92)	72.77 (68.76–76.45)	71.10 (66.92–74.95)	95.87† (94.78–96.74)	91.20 (90.02–92.25)	92.02 (90.91–93.00)	94.85 (93.62–95.85)	91.97† (90.54–93.24)	86.26 (84.64–87.77)	88.30 (86.82–89.66)	89.41 (87.75–90.92)
64.54† (61.36–67.60)	58.06 (55.03–61.03)	63.70† (60.80–66.51)	57.64 (54.30–60.92)	87.54† (85.83–89.06)	83.45 (81.86–84.93)	86.72 (85.21–88.10)	83.70 (81.88–85.37)	79.58† (77.54–81.52)	74.97 (72.97–76.90)	78.96 (77.12–80.71)	74.78% (72.52–76.96)
92.25† (90.20–93.90)	83.72 (81.48–85.73)	91.49† (89.66–93.03)	85.85 (83.27–88.08)	82.95† (80.86–84.86)	80.33 (78.30–82.21)	86.00† (84.20–87.62)	77.76 (75.54–79.84)	87.00† (85.27–88.60)	81.87 (80.07–83.57)	88.43† (86.97–89.77)	81.20 (79.14–83.15)

^†^ higher value comparing analogue versus digital as well as Group A versus B

### Linear mixed model: Treatment, period and interaction effects

Volunteers using the analogue system performed significantly more triages than those using the digital system (p = 0.004). The number of under-triages was significantly higher with the analogue system (lower under-triage rate; p = 0.001). Regarding the four categories shown in Table 7, no significant effects were found for the period and interaction effect. Excluding the interaction or one of the covariates (treatment effect or period effect), only minor differences occurred. As neither period effects nor their interaction with treatment effects reached statistical significance, no indication for relevant carry-over-effects was found.

**Table 7:** Estimations of fixed effects based on linear mixed-effect considering random effects.

		Estimated (95% Confidence interval)	P-value
Triage	Treatment	-5.76 (-9.63–-1.89)	0.004*
	Period	-1.56 (-5.42–2.30)	0.420
	Treatment/Period	-0.01 (-0.03–-0.01)	0.219
Correct triage	Treatment	-1.61 (-4.79–1.57)	0.313
	Period	-1.83 (-5.01–1.34)	0.251
	Treatment/Period	0.00 (-0.01–0.02)	0.762
Over-triage	Treatment	-1.02 (-2.84–0.79–0.79)	0.261
	Period	0.12 (-1.69–1.94)	0.890
	Treatment/Period	-0.00 (-0.01–0.00)	0.241
Under-triage	Treatment	-3.229 (-4.20–-2.6)	<0.001*
	Period	0.12 (-0.85–1.10)	0.799
	Treatment/Period	0.00 (-0.00–0.01)	0.392

Treatment = triage with the analogue or digital system, with the reference category of the analogue system

Period = first and second cycle, with the reference category of the first cycle

Treatment*Period = combination of treatment and period

* Significant < = 0.05

## Discussion

The study aimed to assess the results and training effects of participants’ triage using an analogue and digital documentation system for MCI at sea. The results show that the number of triages comparing the analogue and digital recording system were different. The digital system has a slightly higher reliability and validity than the analogue triage system.

Comparing the data collected in two other studies on the use of an analogue and digital system in triage, the studies achieve results similar to those in the presented study [26,27]. A study as part of the evaluation of a triage app for doctors in a preclinical setting showed that more accurate results can be achieved using analogue documentation [26]. According to a study on the use of an analogue and digital triage system in an emergency room, triage with the digital system achieved more correct results than triage with the analogue system [27].

In contrast to the lower number of triages with the digital system, the comparison of an analogue versus a digital triage system for bio terroristic disasters showed a significantly shorter triage duration for the digital system in a MANV scenario [28]. These might have occurred due to the fact that in the presented study the triage was carried out by non-medical personnel. From an organisational point of view the multifocal screening for several teams with electronically-coupled bundling of the results as well as real-time connection to a coordination point (at sea or on land) improve the benefit of a faster response to the event.

In this study, the unweighted kappa from the analogue triage system indicate a moderate external reliability regarding the interpretation of Landis and Koch [22]. With the digital triage system, substantial agreement was reached. Similar results were found in the comparison of a computerized decision tool and a memory-based triage system of an emergency department, in which the analogue or memory-based triage system showed lower Kappa results than the digital [21].

Compared to the results of a literature review by Wulp et al., the weighted Kappa resulted in fifteen triage evaluations between 0.27 and 0.87 in emergency wards [21]. In that study, nurses, experts and researchers conducted the triage, while non-medical professionals were involved in our triage exercise, reflecting the situation aboard a ship.

The calculation of Cronbach’s Alpha was used to measure the internal consistency of a scale [19]. There are different opinions in the literature, in which Cronbach’s Alpha is considered sufficient to assume an internally consistent scale. Schmitt [20] refers to 0.7 as the desired value. In comparison, Blanz [29] suggested the evaluation of the Cronbach’s Alpha in six categories from bad to possibly unnecessary items. According to Schmitt [20], in this study, all Cronbach’s alpha values show an internally consistent scale. Referring to the evaluation of Blanz [29], the analogue and digital results can be assumed to show “excellent” consistency. Overall, the digital system presented greater reliability.

In general, high specificity is well suited to prove a correct triage result. Tests with high sensitivity are appropriate to exclude a triage result [30,31]. There are only a few publications that used with validity measurements to evaluate the health status of patients in the pre-hospital setting [8,31,32], especially concerning the STaRT-algorithm [8]. In contrast to a previous study [6], the triage of red patients in this study was shown to have lower sensitivity but higher specificity and, compared to the study by Bhalla [30], equal sensitivity and lower specificity. In practice, this means that the identification of red patients was lower in our study, especially with the analogue system. This might be a problem, because the identification of life-threateningly, severely injured patients is a key for triage [3]. Nevertheless, in terms of data interpretation, it must be borne in mind that there were only two red patients in the study of Kahn et al. [8] and the triage was carried out by medical professionals, while the present triage exercise involved a high number of red patients and focused on non-medical personnel. Two of the studies [8,30] reached lower sensitivity and specificity for yellow and green patients than was the case in the present study. Thus, the identification of green patients in our study was more correct, specifically using the digital system. In practical application, correctly identifying green patients to a high degree demonstrates the potential to better allocate the few personnel available to patients with major injuries.

To the authors’ knowledge, there are no comparable publications regarding the predictive value of analogue and digital triage systems for MCI onshore. The positive and negative predictive values of our study were higher in all categories compared to the literature of triage evaluations ashore [8,30]. Only the results for the red patients demonstrated a lower positive predictive value [8] and a negative predictive value of the yellow patients [30].

As Table 7 illustrated, no significant pattern of carry-over effect between triage cycles/ periods occurred. Treatment priorization (triage system) and period (exercise one and two) had no combined effect on training. Only treatment with the analogue system showed a significant effect regarding the number of triage and the number of under-triages.

The number of triage and correct triage varies depending with which triage system the triage started. Group A, which started with the analogue system, shows better results in triage and correctness of triage. Taking a close look at the entire evaluation between digital and analogue, the following pattern appears. With the analogue system, a higher number of triage and transport prioritizations is carried out than with the digital system. The more accurate results are achieved with the digital system. This is not a contradiction. After a systematic review, computer-based and regular training measures can help to improve knowledge about disaster cases for providers outside the hospital [31]. In addition, the variability of the form of teaching is a decisive productivity factor in the area of learning [32].

A result for the concept of triage training is to increase the number of triage and correct triage. Therefore, triage exercises might be more effective with an analogue system, since the number of the triage and the correct triage results is higher in Group A and the analogue triage (Table 3). If the trainees are familiar with the system of triage, the triage result with the digital system might be a suitable system to improve significant the number of correct triages. These results confirm the outcome of a study comparing the computer versus analogue triage system in a live disaster through nurses and physicians. Which come to the result that computer triage might be most efficient for persons familiar with the triage-system [33].

### Limitations

This prospective study was conducted with a small number of participants not working onboard females were overrepresented, which isn’t the regular situation/ fact onboard. This is especially important since the gender aspect has an impact on patient priority [34]. Although the gold standard was evaluated by three different experienced physicians in emergency medicine, it may be a potential source of variation in the analyses. Differences between the triage of real and simulated patients may occur. The time for triage exercise could be an influencing factor. With the digital triage system, the example patients had to be marked with a paper with the triage category. This is an additional time consuming factor which could be accounted in the study design and can explain to some extend the lower number of triages with the digital system. However, a time limit has been set to simulate the time pressure during a MCI and taking the timeframe for the triage with the STaRT-algorithm into account [8]. The limitation of crossover studies is the need for a washout period between study phases, which might have been too short. Therefore, a training effect could result and could lead to a bias with a superior result in the examined triage tool used later on. A limitation relative to data collection occurred by encoding two codes. Hence, not all triage results could be considered in the evaluation. To the best of our knowledge, there is a lack of comparable data of other studies with volunteers or ship crew members regarding triage in the medical literature.

## Conclusion

This prospective study gives reliable and valid results comparing a digital with an analogue triage system for a MCI at sea. Significant differences were found for the number of triages and the number of under-triaged patients between the analogue and digital system, while the number of correct and over-triaged patients was similar in both systems. The results of the study tend to show that the digital system has higher reliability and validity than the analogue system. On the other hand, the number of triages carried out within a given time was higher with the analogue system, which is an important factor for the survival of severely injured patients. The results of the current study offer pioneer evidence for the training of triage for MCI at sea. Even though, there are no consistent data regarding training methods, first advices regarding the use of the analogue system for the basic triage exercises and the digital system for the advanced exercises, can be suggested. One of the influencing factors for the difference between the systems might be the practical applicability of the system. The literature contained no further evidence for the use of triage systems for MCIs at sea, even though mis-triage in MCIs at sea has serious consequences for patients, due to the scarcity of resources. Hence, further studies are necessary, especially the use of triage systems in training situations as well as during MCIs at sea, to develop and evaluate easily applicable triage tools for ship crews.

## Supporting information

S1 Fig Information about the analogue and digital triage system. (PDF)

S2 Fig Data cross-over-study. (PDF)

S1 Table (DOCX)
