# Supplementary material for: Digital versus analogue record systems for mass casualty incidents at sea—Results from an exploratory study
Source: PLoS One. 2020 Jun 5;15(6):e0234156. doi: 10.1371/journal.pone.0234156 (PMC7274416; doi:10.1371/journal.pone.0234156)

Analogue triage system Lübecker Dokumentationssystem für den Großunfall (LüDoG)

Data available under: http://www.luedog.de/

Digital triage system Transport organization for patients at sea (TOPaS)

Registering and triage of the patients

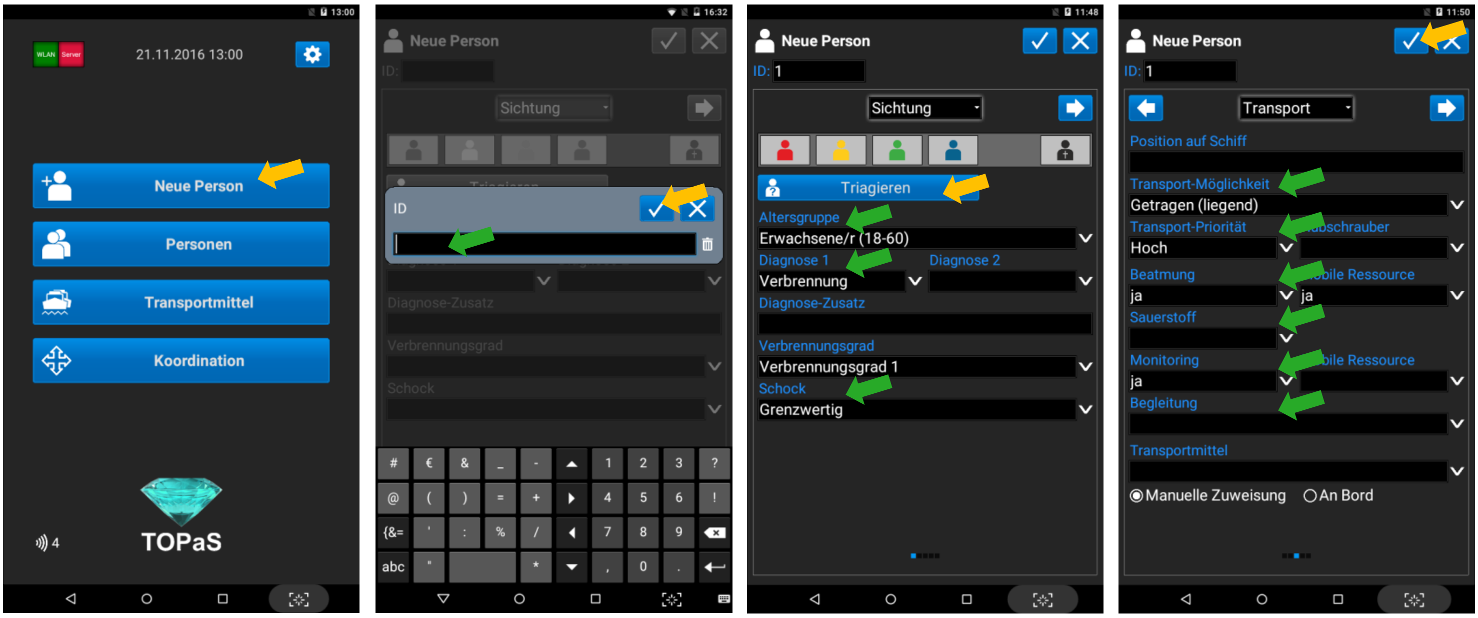

Assignment of patients to the transportation vehicle

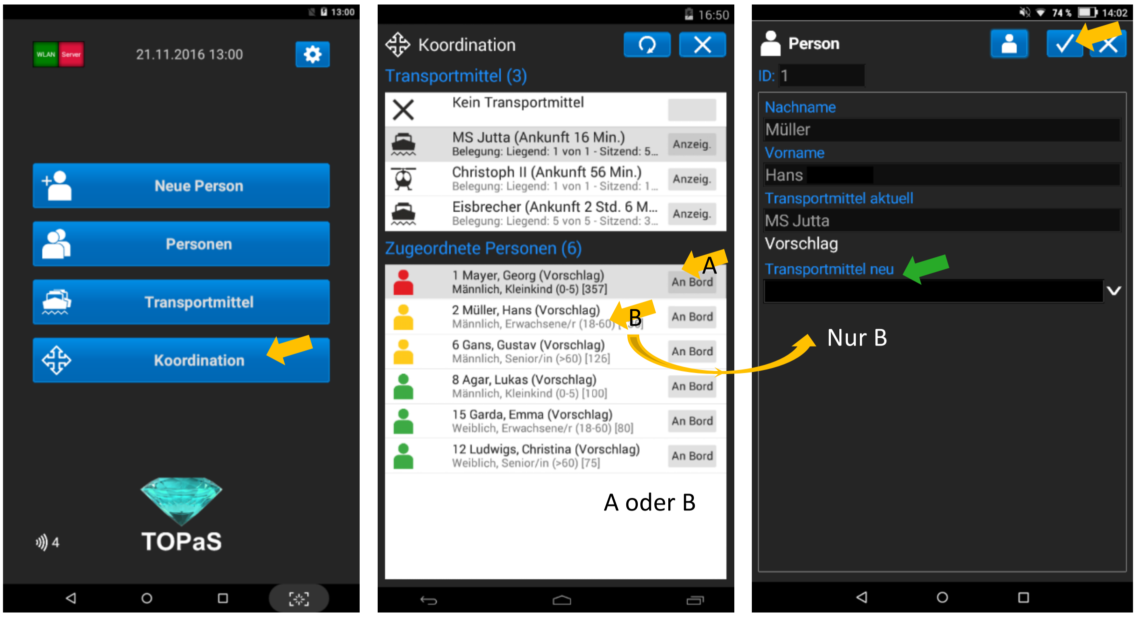

Supplement: S1 Fig — (PDF) [file pone.0234156.s001.pdf]
