# Supplementary material for: Digital versus analogue record systems for mass casualty incidents at sea—Results from an exploratory study
Source: PLoS One. 2020 Jun 5;15(6):e0234156. doi: 10.1371/journal.pone.0234156 (PMC7274416; doi:10.1371/journal.pone.0234156)
Supplement: S2 Fig — (PDF) [file pone.0234156.s002.pdf]

Participants ID	Order of triage (0=analogue-digital; 1=digital-analogue)	Total number of triage	Number of digital triage	Number of analogue triage	Number of correct digital triage	Number of correct analogue triage	Number of digital over-triage	Number of analogue over-triage	Number of digital under-triage	Number of analogue under-triage
1	0	92	46	46	27	33	16	12	3	1
2	0	86	39	47	24	23	10	19	5	5
3	0	87	44	43	21	17	19	18	4	8
4	0	91	41	50	41	33	0	13	0	4
5	0	56	37	19	27	10	6	2	4	7
6	0	75	27	48	23	38	1	2	3	8
7	0	90	40	50	37	35	2	3	1	12
8	0	93	45	48	38	35	3	9	4	4
9	0	100	50	50	46	39	4	3	0	8
10	0	99	49	50	33	30	13	14	3	6
11	0	61	19	42	17	32	1	3	1	7
12	0	56	29	27	29	19	0	2	0	6
13	0	88	41	47	33	26	3	11	5	10
14	0	96	48	48	37	36	8	9	3	3
15	0	93	44	49	31	41	9	4	4	4
16	0	96	49	47	49	33	0	8	0	6
17	0	98	50	48	46	39	3	4	1	5
18	0	79	29	50	22	34	2	8	5	8
19	0	53	31	22	21	14	8	3	2	5
20	0	94	44	50	42	43	2	0	0	7
21	0	92	48	44	44	40	2	2	2	2
22	0	83	35	48	28	40	1	3	6	5
23	0	99	47	52	28	38	19	8	0	6
24	0	92	40	52	37	47	1	2	2	3
25	1	67	46	21	35	13	7	2	4	6
26	1	96	49	47	39	30	8	9	2	8
27	1	69	22	47	19	39	1	5	2	3
28	1	57	23	34	21	24	2	7	0	3
29	1	81	48	33	34	23	13	6	1	4
30	1	77	44	33	32	22	11	8	1	3
31	1	48	20	28	14	18	6	9	0	1
32	1	44	24	20	18	15	4	2	2	3
33	1	55	24	31	24	20	0	1	0	10
34	1	55	20	35	15	31	5	4	0	0
35	1	63	31	32	25	26	6	0	0	6
36	1	60	28	32	23	16	1	13	4	3
37	1	59	28	31	14	18	13	7	1	6
38	1	67	22	45	19	33	0	10	3	2
39	1	67	29	38	23	26	4	5	2	7
40	1	72	25	47	23	33	2	7	0	7
41	1	64	16	48	10	30	4	10	2	8
42	1	42	17	25	13	15	3	9	1	1
43	1	73	25	48	20	34	5	5	0	9
44	1	71	26	45	23	37	3	3	0	5
45	1	44	21	23	10	20	11	1	0	2
46	1	71	24	47	18	28	4	10	2	9
47	1	46	24	22	20	20	3	2	1	0
48	1	48	23	25	15	18	2	6	6	1

	Total	Analogue	Digital	Group A	Group B
Goldstandard triage red	719	397	322	418	301
Goldstandard triage yellow	1079	582	497	622	457
Goldstandard triage green	1747	935	812	1009	738
**Sum**	**3545**	**1914**	**1631**	**2049**	**1496**
Triage red	739	392	347	397	342
Triage yellow	1203	639	564	693	510
Triage green	1582	872	710	943	639
Triage black	21	11	10	16	5
**Sum**	**3545**	**1914**	**1631**	**2049**	**1496**
		0			
Correct triage	2652	1364	1288	1556	1096
Incorrect triage	893	550	343	493	400
**Sum**	**3545**	**1914**	**1631**	**2049**	**1496**
Triage red correct	532	263	269	287	245
Triage red incorrect	207	129	78	110	97
Triage yellow correct	735	371	364	433	302
Triage yellow incorrect	468	268	200	260	208
Triage green correct	1385	730	655	835	550
Triage green incorrect	197	142	55	108	89
Triage black correct	0	0	0	0	0
Triage black incorrect	21	11	10	16	5
**Sum**	**3545**	**1914**	**1631**	**2049**	**1496**
