# Supplementary material for: Digital versus analogue record systems for mass casualty incidents at sea—Results from an exploratory study
Source: PLoS One. 2020 Jun 5;15(6):e0234156. doi: 10.1371/journal.pone.0234156 (PMC7274416; doi:10.1371/journal.pone.0234156)
Supplement: S1 Table — (DOCX) [file pone.0234156.s003.docx]

|  | | Estimation | Standard-Error | Degree of freedom | T-value | P-value | Confidence intervall | |
| --- | --- | --- | --- | --- | --- | --- | --- | --- |
|  |  |  |  |  |  |  | Lower bound | Upper bound |
| Triage | Treatment | -5,763 | 1,921 | 45,512 | -3,000 | 0,004 | -9,631 | -1,895 |
|  | Periode | -1,563 | 1,918 | 45,441 | -0,815 | 0,420 | -5,425 | 2,300 |
|  | Treatment*Periode | -0,012 | 0,009 | 73,393 | -1,241 | 0,219 | -0,031 | 0,007 |
| Correct triage | Treatment | -1,611 | 1,580 | 45,417 | -1,020 | 0,313 | -4,791 | 1,570 |
|  | Periode | -1,833 | 1,577 | 45,352 | -1,163 | 0,251 | -5,009 | 1,342 |
|  | Treatment*Periode | 0,002 | 0,008 | 69,606 | 0,304 | 0,762 | -0,013 | 0,018 |
| Over-triage | Treatment | -1,025 | 0,901 | 45,495 | -1,138 | 0,261 | -2,839 | 0,788 |
|  | Periode | 0,125 | 0,899 | 45,418 | 0,139 | 0,890 | -1,686 | 1,936 |
|  | Treatment*Periode | -0,005 | 0,004 | 77,106 | -1,181 | 0,241 | -0,014 | 0,004 |
| Under-triage | Treatment | -3,229 | 0,490 | 92,000 | -6,594 | 0,000 | -4,202 | -2,256 |
|  | Periode | 0,125 | 0,489 | 92,000 | 0,256 | 0,799 | -0,846 | 1,096 |
|  | Treatment*Periode | 0,002 | 0,002 | 92,000 | 0,859 | 0,392 | -0,002 | 0,006 |
